# Supplementary material for: Predicting visual function by interpreting a neuronal wiring diagram
Source: Nature. 2024 Oct 2;634(8032):113–23. doi: 10.1038/s41586-024-07953-5 (PMC11446822; doi:10.1038/s41586-024-07953-5)
Supplement: Supplementary file 6 — CRF and ERF predictions for individual TmY4 and TmY9 cells. Analogous to Supplementary Data 3, but for TmY target types. Shown are the top four monosynaptic pathways, the strongest pathway passing through each of the top ten intermediary types (ranking from Extended Data Fig. 7), and the trisynaptic pathway Tm1–TmY–Dm3–TmY (see the section entitled Prediction of spatial normalization). [file 41586_2024_7953_MOESM6_ESM.zip › DataS4/TmY9qΓèÑ/720575940636215408.pdf]

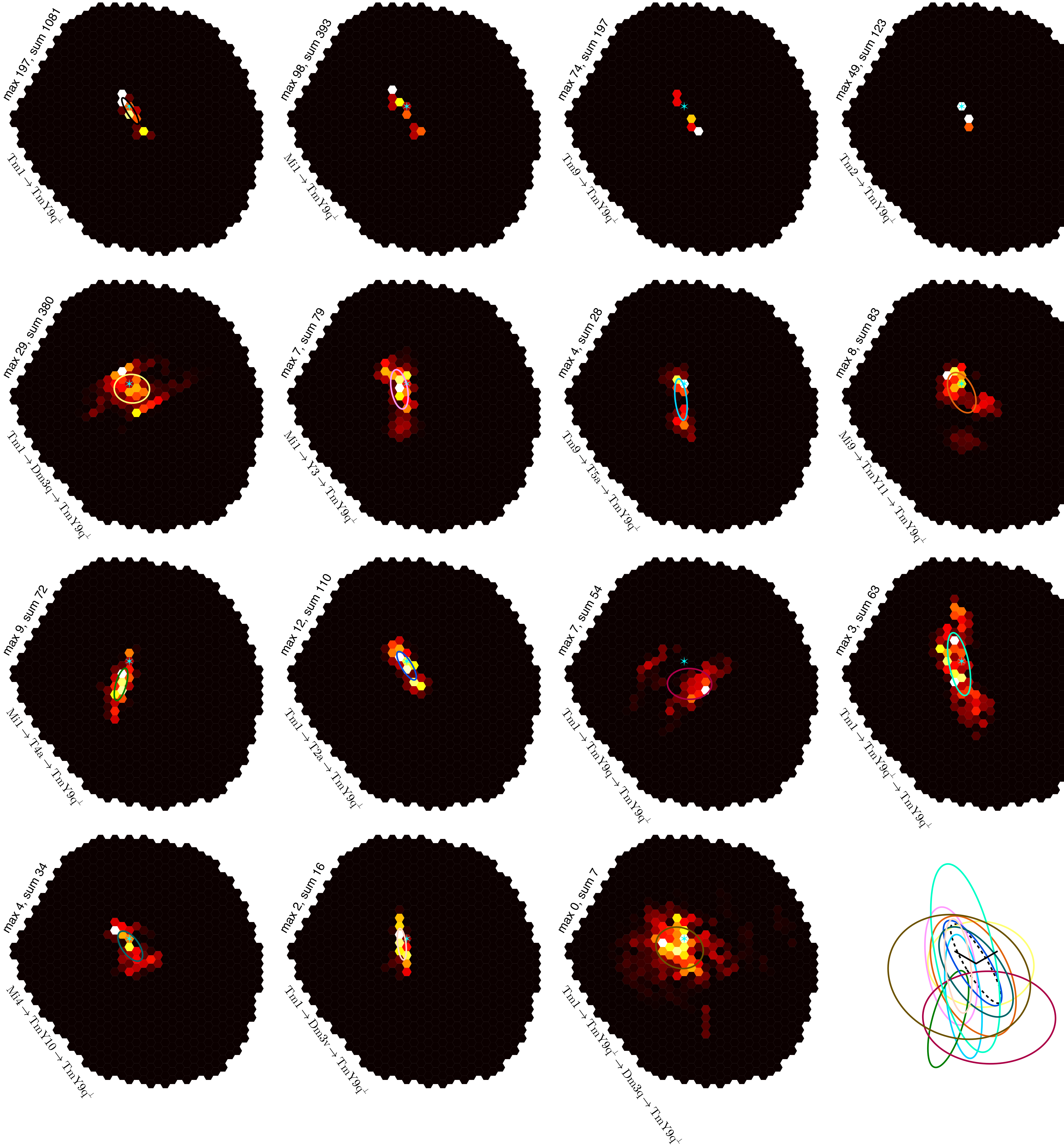

max 197, sum 1081
Tm1 → TmY9q⊥
max 98, sum 393
Mi1 → TmY9q⊥
max 74, sum 197
Tm9 → TmY9q⊥
max 49, sum 123
Tm2 → TmY9q⊥
max 29, sum 380
Tm1 → Dm3q → TmY9q⊥
max 7, sum 79
Mi1 → Y3 → TmY9q⊥
max 4, sum 28
Tm9 → T5a → TmY9q⊥
max 8, sum 83
Mi9 → TmY11 → TmY9q⊥
max 9, sum 72
Mi1 → T4a → TmY9q⊥
max 12, sum 110
Tm1 → T2a → TmY9q⊥
max 7, sum 54
Tm1 → TmY9q → TmY9q⊥
max 3, sum 63
Tm1 → TmY9q⊥ → TmY9q⊥
max 4, sum 34
Mi4 → TmY10 → TmY9q⊥
max 2, sum 16
Tm1 → Dm3v → TmY9q⊥
max 0, sum 7
Tm1 → TmY9q⊥ → Dm3q → TmY9q⊥
